# Supplementary material for: A machine learning Automated Recommendation Tool for synthetic biology
Source: Nat Commun. 2020 Sep 25;11:4879. doi: 10.1038/s41467-020-18008-4 (PMC7519645; doi:10.1038/s41467-020-18008-4)
Supplement: Supplementary file 1 — Supplementary Information [file 41467_2020_18008_MOESM1_ESM.pdf]

Supplementary Information

ART: a machine learning Automated Recommendation Tool for synthetic biology

Tijana Radivojević,[†,‡] Zak Costello,[¶,†,‡] Kenneth Workman,[†,‡,§] and Hector Garcia Martin[*,¶,†,‡,‖]

†*DOE Agile BioFoundry, Emeryville, CA, USA.*

‡*Biological Systems and Engineering Division, Lawrence Berkeley National Laboratory, Berkeley, CA, USA.*

¶*Biofuels and Bioproducts Division, DOE Joint BioEnergy Institute, Emeryville, CA, USA.*

§*Department of Bioengineering, University of California, Berkeley, CA, USA*

‖*BCAM, Basque Center for Applied Mathematics, Bilbao, Spain.*

E-mail: hgmartin@lbl.gov

Contents

Supplementary Methods **4**

Mathematical Methods 4

Markov Chain Monte Carlo sampling 4

Expected value and variance for ensemble model 4

Related work and novelty of our ensemble approach 5

Input space set $\mathcal{B}$ 7

Success probability calculation 7

Multiple response variables 9

Implementation 10

Modules 10

Importing a study 11

Running ART 11

Documentation and testing 14

Supplementary Notes **16**

Designing optimal experiments for machine learning 16

Supplementary Figures and Tables **18**

Figures

1 Code structure 10
2 EDD-style file example 12
3 Naive vs systematic initial data set performance 19
4 Synthetic data MAE 20
5 ART output example 21
6 Linalool and geraniol predictions 22
7 Hopless beer proteomics PCA 23
8 ART for dodecanol, pathway #2 24
9 ART for dodecanol, pathway #3 25

Tables

1 Bayesian-based ensemble modeling approaches comparison 7
2 `Line Name` examples 12
3 ART input parameters 13
4 Dodecanol project pathway designs 19

Supplementary Methods

Mathematical Methods

Markov Chain Monte Carlo sampling

The posterior distribution $p(\boldsymbol{\theta}|\mathcal{D})$ (probability that the parameters $\boldsymbol{\theta}$ fit the data $\mathcal{D}$, used in Eq. 3) is obtained by applying Bayes' formula, i.e. it is defined through a prior $p(\boldsymbol{\theta})$ and a likelihood function $p(\mathcal{D}|\boldsymbol{\theta})$ as

$$p(\boldsymbol{\theta}|\mathcal{D}) \propto p(\mathcal{D}|\boldsymbol{\theta})p(\boldsymbol{\theta}).$$

We define the prior to be $p(\boldsymbol{\theta}) = p(\boldsymbol{w})p(\sigma)$, where $p(\boldsymbol{w})$ is a Dirichlet distribution with uniform parameters, which ensures the constraint on weights (i.e. they all add to one) is satisfied, and $p(\sigma)$ is a half normal distribution with mean and standard deviation set to 0 and 10, respectively. The likelihood function follows directly from Eq. (2) as

$$p(\mathcal{D}|\boldsymbol{\theta}) = \prod_{n=1}^{N} p(y_n|\mathbf{x}_n, \boldsymbol{\theta}), \quad p(y_n|\mathbf{x}_n, \boldsymbol{\theta}) = \frac{1}{\sigma\sqrt{2\pi}} \exp\left\{-\frac{(y_n - \boldsymbol{w}^T\mathbf{f}(\mathbf{x}_n))^2}{2\sigma^2}\right\}.$$

Expected value and variance for ensemble model

From Eq. (2), we can easily compute the expected value

$$\mathbb{E}(y) = \mathbb{E}(\boldsymbol{w}^T\mathbf{f} + \varepsilon) = \mathbb{E}(\boldsymbol{w})^T\mathbf{f} \tag{1}$$

and variance

$$\mathrm{Var}(y) = \mathbf{f}^T\mathrm{Var}(\boldsymbol{w})\mathbf{f} + \mathrm{Var}(\varepsilon) \tag{2}$$

of the response, which will be needed for are used in the optimization phase in order to create the surrogate function $G(\mathbf{x})$ (Eq. 5). The expected value and variance of $\boldsymbol{w}$ and ε are estimated through sample mean and variance using samples from the posterior distribution

$p(\boldsymbol{\theta}|\mathcal{D})$.

Please note that although we have modeled $p(y|\mathbf{x}^*, \boldsymbol{\theta})$ to be Gaussian (Eq. 2), the predictive posterior distribution $p(y|\mathbf{x}^*, \mathcal{D})$ (Eq. 3) is not Gaussian due to the complexity of $p(\boldsymbol{\theta}|\mathcal{D})$ arising from the data and other constraints.

It is important to note that our modeling approach provides quantification of both epistemic and aleatoric uncertainty, through the first and second terms in Suppl. Eq. (2), respectively. Epistemic (systematic) uncertainty accounts for uncertainty in the model and aleatoric (statistical) uncertainty describes the amount of noise inherent in the data.[1] While epistemic uncertainty can be eventually explained away given enough data, we need to model it accurately in order to properly capture situations not encountered in the training set. Modeling epistemic uncertainty is therefore important for small data problems, while aleatoric uncertainty is more relevant for large data problems. In general, it is useful to characterize the uncertainties within a model, as this enables understanding which uncertainties have the potential of being reduced.[2]

Related work and novelty of our ensemble approach

Our ensemble approach is based on stacking[3]—a method where different ensemble members are trained on the same training set and whose outputs are then combined, as opposed to techniques that manipulate the training set (e.g. bagging[4]) or those that sequentially add new models into the ensemble (e.g. boosting[5]). Different approaches for constructing ensemble of models using the Bayesian framework have been already considered. For example, Bayesian Model Averaging (BMA)[6] builds an ensemble model as a linear combination of the individual members in which the weights are given by the posterior probabilities of models. The weights therefore crucially depend on marginal likelihood under each model, which is challenging to compute. BMA accounts for uncertainty about which model is correct but assumes that only one of them is, and as a consequence, it has the tendency of selecting the one model that is the closest to the generating distribution. Agnostic Bayesian learning of

ensembles[7] differs from BMA in the way the weights are calculated. Instead of finding the best predictor from the model class (assuming that the observed data is generated by one of them), this method aims to find the best predictor in terms of the lowest expected loss. The weights are calculated as posterior probability that each model is the one with the lowest loss. Bayesian model combination (BMC)[8] seeks the combination of models that is closest to the generating distribution by heavily weighting the most probable combination of models, instead of doing so for the most probable one. BMC samples from the space of possible ensembles by randomly drawing weights from a Dirichlet distribution with uniform parameters. The Bayesian Additive Regression Trees (BART)[9] method is one of the homogeneous ensemble approaches. It models the ensemble as a (nonweighted) sum of regression trees whose parameters, and ensemble error standard deviation, are defined thought their posterior distributions given data and sampled using MCMC. Yao et al.[10] suggest a predictive model in terms of a weighted combination of predictive distributions for each probabilistic model in the ensemble. This approach can be seen as a generalization of stacking for point estimation to predictive distributions.

All of these models, except of BMC and our model, have weights being point estimates, obtained usually by minimizing some error function. In contrast, we define them as random variables, and in contrast to BMC, our weights are defined through full joint posterior distribution given data. BMC is the closest in design to our approach, but it was formulated only in the context of classifiers. Only BART does include a random error term in the ensemble, apart from our model. Unlike BMA, BMC or models of Yao et al.[10], Chipman et al.[9], our approach does not require that the predictors are themselves probabilistic, and therefore can readily leverage various scikit-learn models. The main differences are summarized in Supplementary Table 1.

Although our model has some features that have been previously considered in the literature, the approach presented here is, to the best of our knowledge, novel in the fact that the metalearner is modeled as a Bayesian linear regression model, whose parameters are

Supplementary Table 1: Feature differences between Bayesian based ensemble modeling approaches.

Method	Weighted average	Probabilistic base models	Probabilistic weights	Regression	Classification	Ensemble error term
BMA[6]	✔	✔	✘	✔	✔	✘
BMC[8]	✔	✔	✔✘	✘	✔	✘
BART[9]	✘	✔	✘	✔	✘	✔
Stacking predictive distributions[10]	✔	✔	✘	✔	✔	✘
Agnostic Bayes[7]	✔	✔✘	✘	✔	✔	✘
ART (this work)	✔	✘	✔	✔	✘	✔

inferred from data combined with a prior that satisfies the constraints on the 'voting' nature of ensemble learners.

Input space set $\mathcal{B}$

The bounds for the input space $\mathcal{B}$ for $G(\mathbf{x})$ (Eq. 4) can be provided by the user (see details in the "Implementation" section, Supplementary Table 3). Otherwise, default values are computed from the input data defining the feasible space as:

$$
\begin{aligned}
\mathcal{B} = \{\tilde{\mathbf{x}} \in \mathbb{R}^D | \; L_d - \Delta_d \leq \tilde{x}^d \leq U_d + \Delta_d \;\;, \; d = 1, \ldots, D\} \\
\Delta_d = (U_d - L_d)\epsilon \;\; ; \;\; U_d = \max_{1 \leq n \leq N}(x_n^d) \;\; ; \;\; L_d = \min_{1 \leq n \leq N}(x_n^d) \\
(\mathbf{x}_n, y_n) \in \mathcal{D}, \; n = 1, \ldots, N
\end{aligned} \tag{3}
$$

This restriction of input variables to the set $\mathcal{B}$ reflects our assumption that the predictive models performs accurately enough only on an interval that is enlarged by a factor ϵ around the minimum and maximum values in the data set ($\epsilon = 0.05$ in all calculations).

Success probability calculation

Our probabilistic model enables us to estimate the probability of success for the provided recommendations. Of practical interest are the probability that a single recommendation is successful and the probability that at least one recommendation of several provided is

successful.

Success is defined differently for each of the three cases considered in Eq. (5): maximization, minimization and specification. For maximization, success involves obtaining a response y higher than the success value y^* defined by the user (e.g. the best production so far improved by a factor of 20%). For minimization, success involves obtaining a response lower than the success value y^*. For the specification case, it involves obtaining a response that is as close as possible to the success value y^*.

Formally, we define success for response y through the set $\mathcal{S} = \{y | y \sim p_{\mathcal{S}}(y)\}$, where the probability distribution for success is

$$p_{\mathcal{S}}(y) = \begin{cases} \mathcal{U}(y^*, U) & \text{(maximization case)} \\ \mathcal{U}(L, y^*) & \text{(minimization case)} \\ \mathcal{N}(y^*, \sigma_{y^*}^2) & \text{(specification case),} \end{cases} \tag{4}$$

where $\mathcal{U}$ is the uniform distribution ($\mathcal{U}(a, b) = 1/(b - a)$ if $a < y < b$; 0 otherwise), L and U are its corresponding lower and upper bounds, and $\sigma_{y^*}^2$ is the variance of the normal distribution $\mathcal{N}$ around the target value y^* for the specification case.

The probability that a recommendation succeeds is given by integrating the probability that input $\mathbf{x}^r$ gives a response y (i.e. full predictive posterior distribution from Eq. 3), times the probability that response y is a success

$$p(\mathcal{S}|\mathbf{x}^r) = \int p_{\mathcal{S}}(y) p(y|\mathbf{x}^r, \mathcal{D}) \mathrm{d}y.$$

This success probability is approximated using draws from the posterior predictive distribution as

$$p(\mathcal{S}|\mathbf{x}^r) \approx \begin{cases} \frac{1}{N_s} \sum_{i=1}^{N_s} \mathbb{I}_{\mathcal{S}}(y_i) & \text{(maximization/minimization case)} \\ \frac{1}{N_s} \sum_{i=1}^{N_s} \mathcal{N}(y_i; y^*, \sigma_{y^*}^2) & \text{(specification case)} \end{cases} \tag{5}$$

where $y_i \sim p(y|\mathbf{x}^r, \mathcal{D}), i = 1, \ldots, N_s$, and $\mathbb{I}_{\mathcal{A}}(y) = 1$ if $y \in \mathcal{A}$, 0 if $y \notin \mathcal{A}$.

In case of multiple recommendations $\{\mathbf{x}^r\} \equiv \{\mathbf{x}^r\}_{r=1}^{N_r}$, we provide the probability of success for at least one of the recommendations only for maximization and minimization types of objectives. This probability is calculated as one minus the probability $p(\mathcal{F}|\{\mathbf{x}^r\})$ that all recommendations fail, where

$$p(\mathcal{F}|\{\mathbf{x}^r\}) \approx \frac{1}{N_s} \sum_{i=1}^{N_s} \mathbb{I}_{\mathcal{F}}(\{y_i^r\}), \ \{y_i^r\} \sim p(y|\{\mathbf{x}^r\}, \mathcal{D}), i = 1, \ldots, N_s, r = 1, \ldots, N_r,$$

and the *failure* set $\mathcal{F} = \{\{y^r\}|y^r \notin \mathcal{S}, \forall r = 1, \ldots, N_r\}$ consists of outcomes that are not successes for all of the recommendations. Since the chosen recommendations are not necessarily independent, we sample $\{y_i^r\}$ jointly for all $\{\mathbf{x}^r\}$, i.e. i-th sample has the same model parameters ($w_i, \sigma_i, \varepsilon_{ij} \sim \mathcal{N}(0, \sigma_i^2)$ from Eq. 2) for all recommendations.

Multiple response variables

For multiple response variable problems (e.g. trying to hit a predetermined value of metabolite a and metabolite b simultaneously, as in the case of the hopless beer), we assume that the response variables are conditionally independent given input vector $\mathbf{x}$, and build a separate predictive model $p_j(y_j|\mathbf{x}, \mathcal{D})$ for each variable $y_j, j = 1, \ldots, J$. We then define the objective function for the optimization phase as

$$G(\mathbf{x}) = (1 - \alpha) \sum_{j=1}^{J} \mathbb{E}(y_j) + \alpha \sum_{j=1}^{J} \mathrm{Var}(y_j)^{1/2}$$

in case of maximization, and analogously adding the summation of expectation and variance terms in the corresponding functions for minimization and specification objectives (Eq. 5). The probability of success for multiple variables is then defined as

$$p(\mathcal{S}_1, \ldots, \mathcal{S}_J|\mathbf{x}) = \prod_{j=1}^{J} p(\mathcal{S}_j|\mathbf{x}^r)$$

Future work will address the limitation of the independence assumption and take into account possible correlations among multiple response variables.

Implementation

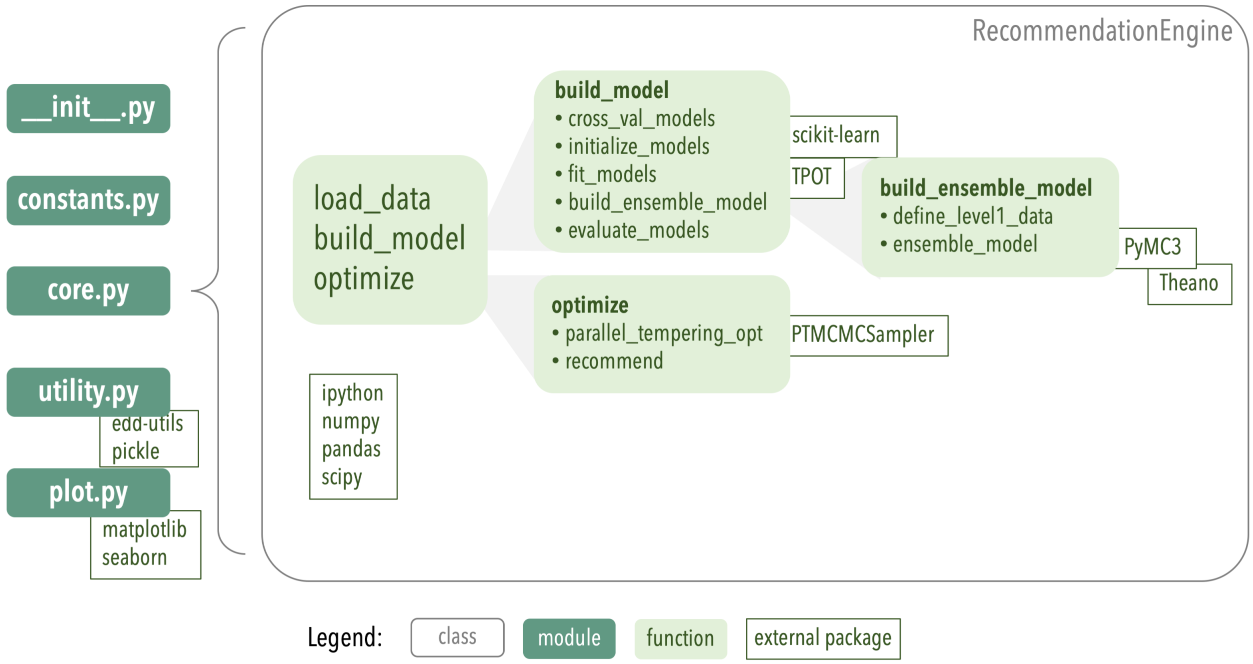

Supplementary Figure 1: The main ART source code structure and dependencies.

Modules

`core.py` is the core module that defines the class `RecommendationEngine` with functions for loading data (into the format required for machine learning models), building predictive models and optimization (Supplementary Figure 1).

The module `constants.py` contains assignments to all constants appearing throughout all other modules. Those include default values for some of the optional user input parameters (Supplementary Table 3), hyperparameters for `scikit-learn` models and simulation setups for `PyMC3` and `PTMCMCSampler` functions.

Module `utilities.py` is a suite of functions that facilitate ART's computations but

can be used independently. It includes functions for loading studies (using EDD through `edd-utils` or directly from files), metrics for evaluation of predictive models, identifying and filtering noisy data, etc.

Module `plot.py` contains a set of functions for visualization of different quantities obtained during an ART run, including functions of relevance to final users (e.g. true vs. predicted values) as well as those providing insights into intermediate steps (e.g. predictive models surfaces, space exploration from optimization, recommendations distributions).

All modules can be easily further extended by future contributors to ART.

Importing a study

Studies can be loaded directly from EDD by calling a function from the `utility.py` module that relies on `edd-utils` package:

```
dataframe = load_study(edd_study_slug=edd_study_slug,edd_server=edd_server)
```

The user should provide the study slug (last part of the study web address) and the url to the EDD server. Alternatively, a study can be loaded from an EDD-style .csv file, by providing a path to the file and calling the same function:

```
dataframe = load_study(data_file=data_file)
```

The .csv file should have the same format as an export file from EDD, i.e. it should contain at least `Line Name` column, `Measurement Type` column with names of input and response variables, and `Value` column with numerical values for all variables (see example in Supplementary Figure 2).

Either approach will return a pandas dataframe containing all information in the study, which can be pre-processed before running ART, if needed.

Running ART

ART can be run by instantiating an object from the `RecommendationEngine` class by:

```
art = RecommendationEngine(dataframe, **art_params)
```

	A	B	C
1	**Line Name**	**Measurement Type**	**Value**
2	B-Mh	MVD1_YEAST	1.7944
3	B-Mh	Q40322_MENSP	5.0071
4	B-Mh	IDI_ECOLI	1.7301
5	B-Mh	ATOB_ECOLI	0.6159
6	B-Mh	Q8LKJ3_ABIGR	0.1409
7	B-Mh	Q9FD87_STAAU	1.9505
8	B-Mh	Q9FD86_STAAU	1.4415
9	B-Mh	KIME_YEAST	0.3235
10	B-Mh	ERG8_YEAST	0.9816
11	B-Ml	MVD1_YEAST	1.129
12	B-Ml	Q40322_MENSP	4.5317

Supplementary Figure 2: Example of an EDD-style file.

The first argument is the dataframe created in the previous step (from an EDD study or data file import). If there is no data preprocessing, the dataframe is ready to be passed as an argument. Otherwise, the user should make sure that the dataframe contains at least the required columns: `Line Name`, `Measurement Type` and `Value`. Furthermore, line names should always contain a hyphen ("`-`") denoting replicates (see Supplementary Table 2), and this character should be exclusively used for this purpose (this point is critical for creating partitions for cross-validation).

Supplementary Table 2: Valid and non valid examples of entries of the `Line Name` column in the dataframe passed to start an ART run.

✔ Valid	✘ Non valid
`LineNameX-1`	`LineNameX1`
`LineNameX-2`	`LineNameX2`
`LineNameX-r1`	`Line-NameX1`
`LineNameX-r2`	`Line-NameX2`
`LineNameX-R1`	`Line-Name-X1`
`LineNameX-R2`	`Line-Name-X2`
...	...

The second argument is a dictionary of key-value pairs defining several required and optional keyword arguments (summarized in Supplementary Table 3) for generation of an `art` object.

Building the model

The level-0 models are first initialized and then fitted through the `_initialize_models`

Supplementary Table 3: ART input parameters. Required parameters are marked with an asterisk.

Name	Meaning
input_var	List of input variables*
bounds_file	Path to the file with upper and lower bounds for each input variable (default None)
response_var	List of response variables*
build_model	Flag for building a predictive model (default True)
cross_val	Flag for performing cross-validation (default False)
ensemble_model	Type of the ensemble model (default 'BW')
num_models	Number of level-0 models (default 8)
recommend	Flag for performing optimization and providing recommendations (default True)
objective	Type of the objective (default 'maximize')
threshold	Relative threshold for defining success (default 0)
target_value	Target value for the specification objective (default None)
num_recommendations	Number of recommendations for the next cycle (default 16)
rel_eng_accuracy	Relative engineering accuracy or required relative distance between recommendations (default 0.2)
niter	Number of iterations to use for $T = 1$ chain in parallel tempering (default 100000)
alpha	Parameter determining the level of exploration during the optimization (value between 0 and 1, default None corresponding to 0)
output_directory	Path of the output directory with results (default ../results/response_var_time_suffix)
verbose	Amount of information displayed (default 0)
seed	Random seed for reproducible runs (default None)

and _fit_models functions respectively, which rely on the scikit-learn and tpot packages. To build the final predictive model, first the level-1 data is created by storing cross-validated predictions of level-0 models into a theano variable that is shared across the functions from the PyMC3 package. Finally, the parameters of the ensemble model are sampled within the function _ensemble_model, which stores the inferred model and traces that are later used for predictive posterior probability calculation, as well as first and second moments from the traces, used for estimation of the first two moments of the predictive posterior distribution using Suppl. Eq. (1)–(2).

By default, ART builds the models using all available data and evaluates the final, ensemble model, as well as all level-0 models, on the same data. Optionally, if specified by the user

through the input flag `cross_val`, ART will evaluate the models on 10-fold cross-validated predictions, through the function `_cross_val_models`. This computation lasts roughly 10 times longer. Evaluating models on new data, unseen by the models, can also be done by calling:

```
art.evaluate_models(X=X_new, y=y_new)
```

Optimization

ART performs optimization by first creating a set of draws from

```
draws = art.parallel_tempering_opt()
```

which relies on the `PTMCMCSampler` package. Here, an object from the class `TargetModel` is created. This class provides a template for and can be replaced by other types of objective functions (or target distributions) for parallel tempering type of optimization, as long as it contains functions defining loglikelihood and logprior calculation (see Eq. 6). Also, the whole optimization procedure may well be replaced by an alternative routine. For example, if the dimension of the input space is relatively small, a grid search could be performed, or even evaluation of the objective at each point for discrete variables. Lastly, out of all draws collected by optimizing the specified objective, ART finds a set of recommendations by

```
art.recommend(draws)
```

which ensures that each recommendation is different from all others and all input data by a factor of γ (`rel_eng_accuracy`) in at least one of the components (see Algorithm 1).

Documentation and testing

The ART source code thoroughly conforms to the syntactic and stylistic specifications outlined in the PEP 8 style guide.[11] Documentation for class methods and utility functions is embedded in docstrings. A brief synopsis of functionality, an optional deeper dive into behavior and use cases, as well as a full description of parameter and return value typing is included in the flexible reStructuredText markup syntax. The documented source code is used in conjunction with the `Sphinx` package to dynamically generate an API reference.

Algorithm 1 Choosing recommendations from a set of samples from the target distribution $\pi(\mathbf{x})$

Input: N_r: number of recommendations
  $\{\mathbf{x}_n\}_{n=1}^{N_s}$: samples from $\pi(\mathbf{x})$ (Eq. 6)
  γ: required relative distance for recommendations (relative engineering accuracy)
  $\mathcal{D}_{\mathbf{x}}$: input variable experimental data
Output: $rec = \{\mathbf{x}^r\}_{r=1}^{N_r}$: set of recommendations
$draws \leftarrow \{\mathbf{x}_n\}_{n=1}^{N_s}$ {remaining draws}
$rec = \emptyset$
while $r = 1, \ldots, N_r$ **do**
  if $draws = \emptyset$ **then**
    $\gamma = 0.8\gamma$ and repeat the procedure
  else
    $\mathbf{x}^r \leftarrow$ a sample from $draws$ with maximal $G(\mathbf{x})$ {$G(\mathbf{x})$ is already calculated}
    if there exists $d \in \{1, \ldots, D\}$ s.t. $|x_d^r - x_d| > \gamma$ for all $\mathbf{x} \in rec \cup \mathcal{D}_{\mathbf{x}}$ **then**
      $rec = \{rec, \mathbf{x}^r\}$
    end if
  end if
  $draws \leftarrow draws \setminus \{\mathbf{x}_n \in draws | G(\mathbf{x}_n) = G(\mathbf{x}^r)\}$
end while
return rec

The code can be built as a local package for testing and other utility, the dependencies and procedure for which are handled by the `setup.py` file in accordance with the Setuptools standard for module installation.

A suite of unit and integration tests were written using the `pytest` library, and are included with the source code under the `tests` directory. The unit testing was designed to target and rigorously validate individual functions in their handling and output of types. Because of the expensive calls to libraries such as `scikit-learn` and `tpot` laden throughout ART's codebase for model training and validation, unit-tested functions were parameterized with the Boolean flag `testing` to replace such calls with dummy data that mimic the responses from library code. The resulting suite of unit tests therefore runs rapidly, quickly evaluating the integrity of type handling and output that ensure the smooth hand-off of values between functions in ART.

Integration tests are likewise implemented with `pytest`, but rather test a more complete

spectrum of expected behavior without silencing calls to library code. Full model training, cross-validation, and evaluation is completed under this test suite, the output of which is validated against known data sets within some reasonable margin to account for stochastic processes.

The instructions to locally run the testing suite can be found in the documentation.

Supplementary Notes

Designing optimal experiments for machine learning

Machine learning is not a magical procedure that can just be sprinkled over any data set to obtain good results: it requires careful experimental planning to meet the requirements that will make it effective. A wrong choice for the initial training data set, for example, can lengthen the learning process significantly (Supplementary Figure 3).

Here, we offer a succinct list of points to consider when planning to use ART (or ML in general) to guide bioengineering:

- **Choose actionable inputs**. Choose as input features for ART (see Fig. 1) experiment variables that can be easily manipulated, because the ML-guided bioengineering procedure will require them to be changed in order to explore the full phase space and "learn" the problem. For example, choose transcriptomics and proteomics over metabolomics: most metabolic engineers know how to engineer their pathway to increase or decrease the amount of a given transcript or protein, whereas deciding what to change to increase e.g. acetyl-coA concentration two-fold is not trivial. However, one cannot take reaching proteomics targets for granted, as shown in the "Improving dodecanol production" section. An alternative is to use variables completely under our control as input, such as the promoters for the five genes used in Zhang et al.[12]. This approach has the advantage that it fully bridges the Learn and Design phases of

the DBTL cycle, but it has the disadvantage that it may not fully explore the protein phase space (e.g. in case all promoters available are weak for a given protein).

- **Decide very carefully how many experiment variables (i.e. input features) you would like to explore.** Choose too many variables (e.g. proteins in the limonene case in the "Improving the production of renewable biofuel" section, or promoters in Zhang et al.[12]) and the corresponding phase space is too large for machine learning to explore in a reasonable amount of DBTL cycles. Choose too few variables and you might miss important configurations for your system. As a *very crude* rule of thumb, you should budget for around ∼100 instances per 5-10 variables.

- **Make sure your experiment variables can be independently acted upon.** If recommendations require protein 1 concentration to be increased two-fold and protein 2 to be decreased by a factor of three to improve production, but a strong promoter for protein 1 also produces an increase in protein 2, it will very complicated to reach the target protein profile. This type of non-target effects were abundantly found in the dodecanol case[13] ("Improving dodecanol production" section). Modular pathway designs that ensure that the full input phase space we can be fully explored are, therefore, highly recommended. Systematic part characterization (e.g. large promoter libraries with a variety of tested relative strengths) are equally important.

- **Design your experiment to start with ∼100 instances for the initial DBTL cycle.** Although we have shown that ART can use less than a hundred instances as starting point and still guide metabolic engineering effectively (e.g. limonene and hoppy beer cases in the "Improving the production of renewable biofuel" and "Brewing hoppy beer without hops by bioengineering yeast" sections), this is by no means a guarantee, and actual successs depends on the complexity of the problem (see the "Using simulated data to test ART" section). By starting with ∼100 instances, one can alleviate the problem of lack of statistical convergence, if predictions are not accurate.

The alternative is a non-predictive model and little understanding whether the problem is lack of data (instances), or other design problems (see the "Improving dodecanol production" section). Consider automating as much of your process as possible to guarantee enough instances.

- **Sample the initial phase space as widely as possible.** Make sure you cover wide ranges for both input and response variables. Include bad (e.g. low production) and intermediate results as well as good ones (e.g. high production). This is the only way that the algorithms can learn to distinguish the inputs needed to reach any of these regimes. The Latin Hypercube[14] that we used for the synthetic data case ("Using simulated data to test ART" section) is a good choice, but by no means the only one.

- **Plan for several DBTL cycles.** ART, and machine learning methods in general, shine when they can dynamically probe your system. While you can get results with two DBTL cycles, they are not comparable to what you get using >5 cycles (Fig. 5 in the main text). If you have a limited budget of, e.g. 100 instances, it is better to start with a strong first cycle and several weaker ones (e.g. 40 instances for cycle 1, then six 10 instance cycles) than the customary two DBTL cycle study (e.g. 60 instances for first cycle, 40 instances for the second one).

- **Consider uncertainty, as well as predicted response, when choosing next steps.** Choose some recommendations with little uncertainty even if the predicted outcome is not so desirable (e.g. low production) to establish trust in the approach. Choose some recommendations with large uncertainty even if the predicted outcome is not desirable so as not to miss unexpected opportunities. Use the α parameter (Eq. 5 in the main text) for this purpose.

Supplementary Figures and Tables

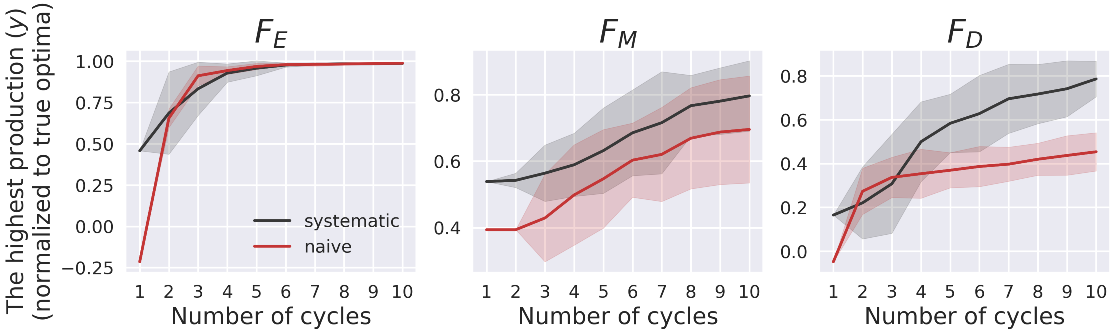

Supplementary Figure 3: **Choosing the initial training data set wisely can increase ART's performance significantly.** The "systematic" approach involves using the Latin Hypercube[14] method to choose starting points in the phase space, whereas the "naive" approach concentrates all starting points in a portion of the whole phase space that spans only 1/10th of the width for each dimension. As in Fig. 5, plots present the results of the simulated metabolic engineering in terms of highest production achieved so far for each cycle, here shown for $D = 10$. The systematic approach outperforms the naive one for the difficult and medium type of functions, whereas both approaches perform similarly for the easy function from cycle 2 onwards (see Figure 5 in the main text). In each plot, lines and shaded areas represent the estimated mean values and 95% confidence intervals, respectively, over 10 repeated runs. Source data are provided as a Source Data file.

Supplementary Table 4: Total number of strains (pathway designs) and training instances available for the dodecanol production study[13] (Fig. 9, Suppl. Figs. 8 and 9). Pathway 1, 2 and 3 refer to the top, medium and bottom pathways in Fig. 1B of Opgenorth et al.[13]. Training instances are amplified by the use of fermentation replicates. Failed constructs (3 in each cycle, initial designs were for 36 and 24 strains in cycle 1 and 2) indicate nontarget, possibly toxic, effects related to the chosen designs. Numbers in parentheses () indicate cases for which no product (dodecanol) was detected.

	Number of **strains**		Number of **instances**	
	Cycle 1	Cycle 2	Cycle 1	Cycle 2
Pathway 1	12	11 (2)	50	39 (6)
Pathway 2	9 (4)	10 (5)	31 (10)	30 (14)
Pathway 3	12	-	35	-
Total	33 (4)	21 (7)	116 (10)	69 (20)

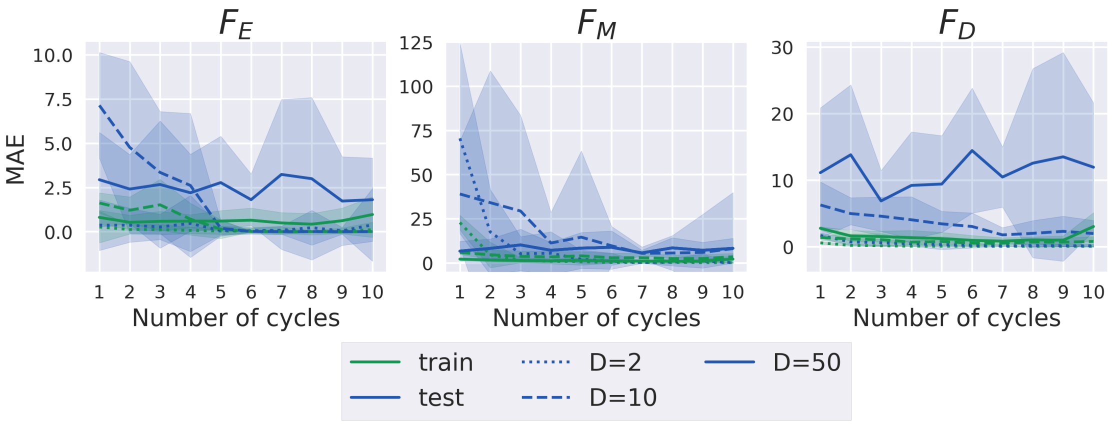

Supplementary Figure 4: **Mean Absolute Error (MAE) for the synthetic data set in Fig. 5.** Synthetic data is obtained from functions of different levels of complexity (see Figure 4 in the main text), different phase space dimensions (2, 10 and 50), and different amounts of training data (DBTL cycles). The training set involves all the strains from previous DBTL cycles. The test set involves the recommendations from the current cycle. MAE are obtained by averaging the absolute difference between predicted and actual production levels for these strains. MAE decreases significantly as more data (DBTL cycles) are added, with the exception of the high dimension case. In each plot, lines and shaded areas represent the estimated mean values and 95% confidence intervals, respectively, over 10 repeated runs. Source data are provided as a Source Data file.

	ATOB_ECOLI	ERG8_YEAST	IDI_ECOLI	KIME_YEAST	MVD1_YEAST	Q40322_MENSP	Q8LKJ3_ABIGR	Q9FD86_STAAU	Q9FD87_STAAU	Limonene
0	0.23	0.49	3.27	1.39	4.28	14.19	4.69	0.43	0.56	119.28
1	0.30	0.45	3.27	1.42	4.12	14.18	4.72	0.44	0.54	119.18
2	0.25	0.30	3.25	1.42	4.28	14.15	4.72	0.42	0.58	119.15
3	0.23	0.23	2.65	1.42	4.09	14.14	4.74	0.44	0.55	119.14
4	0.27	0.67	3.28	1.41	4.14	14.18	4.74	0.44	0.54	119.07
5	0.22	0.37	2.66	1.46	4.28	13.90	4.74	0.44	0.55	119.05
6	0.41	0.44	2.62	1.47	3.97	14.13	4.70	0.42	0.54	118.97
7	0.23	0.45	2.60	1.48	4.11	14.16	4.75	0.43	0.61	118.96
8	0.23	0.21	3.27	1.24	4.23	14.21	4.73	0.44	0.54	118.90
9	0.36	0.34	2.56	1.37	4.16	14.16	4.72	0.42	0.54	118.76

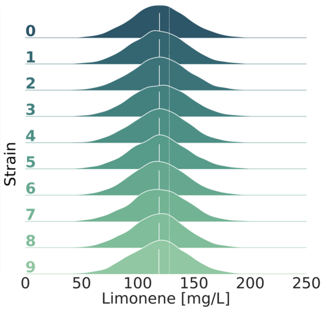

Supplementary Figure 5: **Example for ART's output for the limonene case.** The output comes in the form of recommended inputs for the next cycle (left) and the corresponding probability distribution for the associated response (right) (see the "Improving the production of renewable biofuel" section for further details). Note that the recommendations come in the same form as the input. The inputs in this case are the protein profiles for 9 proteins and, hence, the recommendations come in terms of target protein profiles for the same 9 proteins. The response in this case is the production of limonene, and the second part of the output represents the probability distribution for the limonene production (right). The way to use these recommendations is to 1) find an existing strain, and 2) find the relative protein expression changes from this strain to the recommendations: e.g. protein 1 needs to be 3× more abundant, protein 2 needs to stay roughly the same, protein 3 concentration needs to be zero, protein 4 needs to be halved, etc. Modifying the pathway to achieve these changes is non trivial because tools to predict protein expression from DNA parts can be very inaccurate (see the "Improving dodecanol production" section). An alternative approach that bypasses this problem involves using, as input to ART, the pathway components: e.g. gene promoters as we did in Zhang et al.[12]. Providing the recommendations in the form of DNA parts (e.g. from the MoClo toolkit[15]) must be tailored to each project specifically.

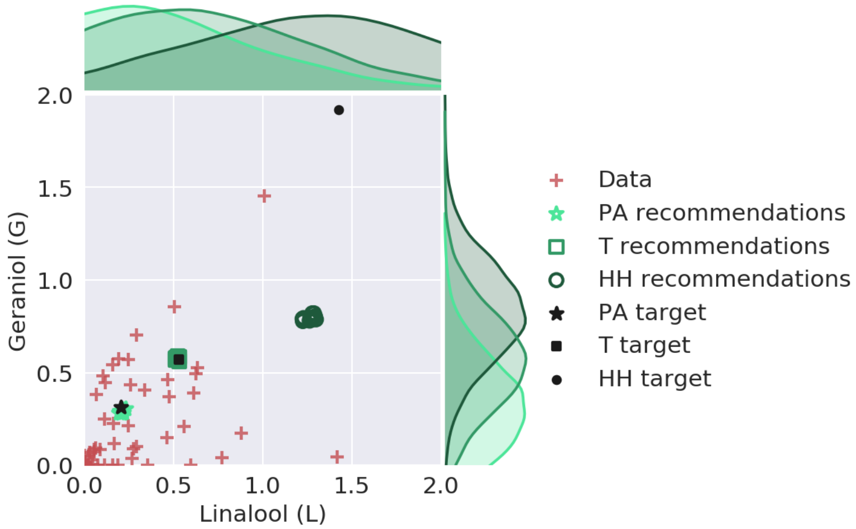

Supplementary Figure 6: **Linalool and geraniol predictions for ART recommendations for each of the beers (Fig. 8), showing full probability distributions (not just averages)**. These probability distributions (in different tones of green for each of the three beers) show very broad spreads, belying the illusion of accurate predictions and recommendations. These broad spreads indicate that the model has not converged yet and that many production levels are compatible with a given protein profile. Source data are provided as a Source Data file.

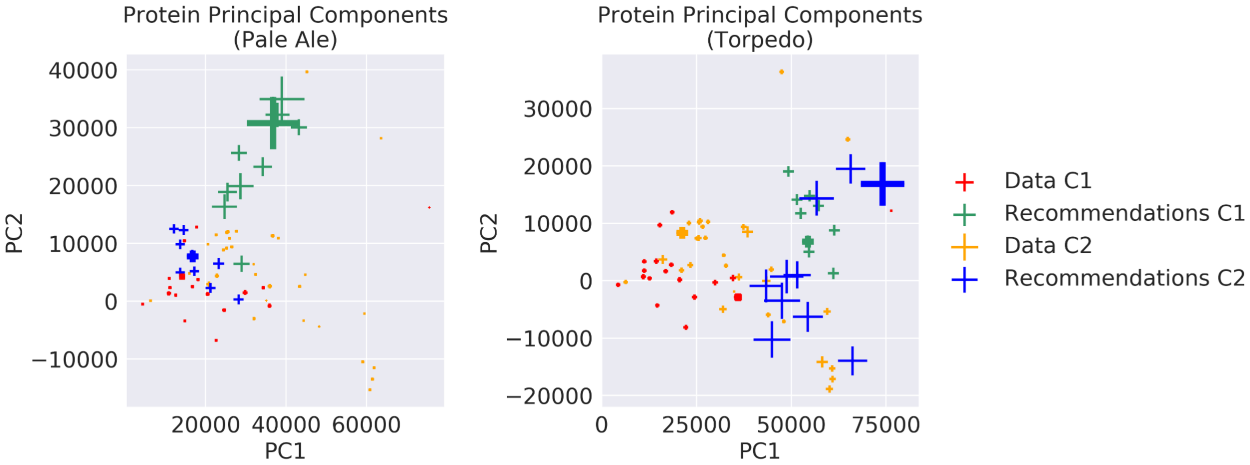

Supplementary Figure 7: **Principal Component Analysis (PCA) of proteomics data for the hopless beer project (Fig. 8)**, showing experimental results for cycle 1 and 2, as well as ART recommendations for both cycles. Cross size is inversely proportional to proximity to L and G targets (larger crosses are closer to target). The broad probability distributions spreads (Supplementary Figure 6) suggest that recommendations will change significantly with new data. Indeed the protein profile recommendations for the Pale Ale changed markedly from DBTL cycle 1 to 2, even though the average metabolite predictions did not (Fig. 8, c) and g)). For the Torpedo case, the final protein profile recommendations overlapped with the experimental protein profiles from cycle 2, although they did not cluster around the closest profile (largest orange cross), concentrating on a better solution according to the model. In any case, despite the limited predictive power afforded by the cycle 1 data, ART produces recommendations that guide the metabolic engineering effectively. For both of these cases, ART recommends exploring parts of the phase space such that the final protein profiles that were deemed close enough to the targets (in orange, see also g) in Fig. 8) lie between the first cycle data (red) and these recommendations (green). In this way, finding the final target (expected around the orange cloud) becomes an interpolation problem, which is easier to solve than an extrapolation one. Source data are provided as a Source Data file.

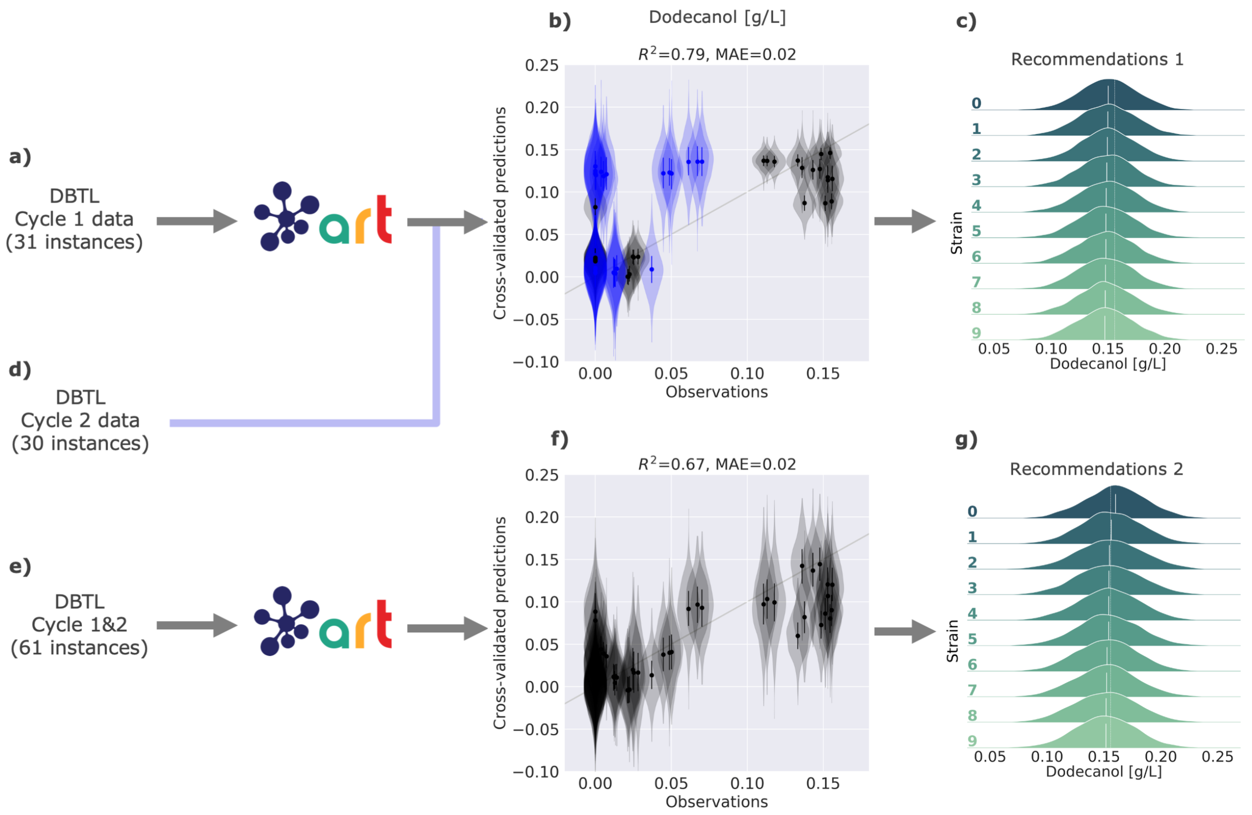

Supplementary Figure 8: **ART's predictive power for the second pathway in the dodecanol production example is very limited.** Although cycle 1 data provide good cross-validated predictions (in black), testing the model with 30 new instances from cycle 2 (in blue) shows limited predictive power and generalizability. As in the case of the first pathway (Fig. 9), combining data from cycles 1 and 2 improves predictions significantly. The points and the violins represent the mean values and the uncertainty in predictions, respectively. R^2 and Mean Absolute Error (MAE) values are only for cross-validated mean predictions. Source data are provided as a Source Data file.

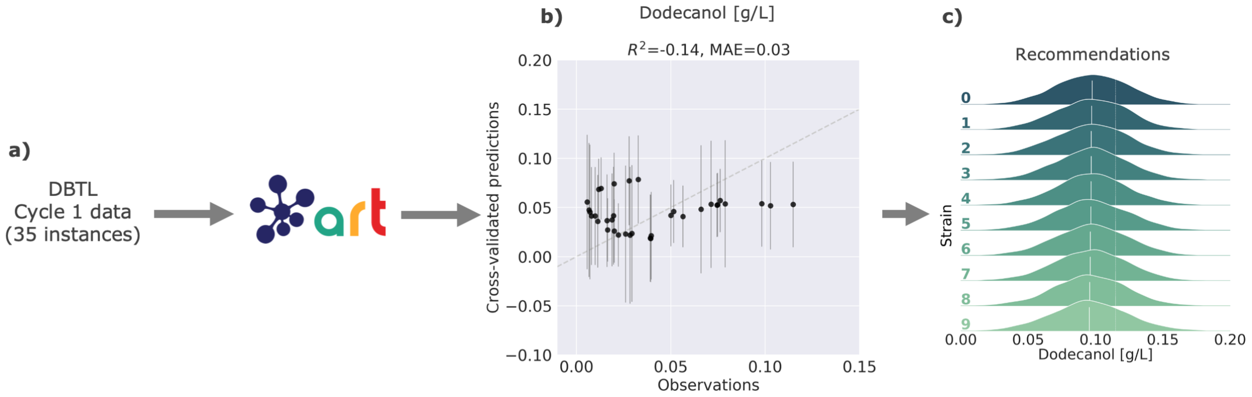

Supplementary Figure 9: **ART's predictive power for the third pathway in the dodecanol production example is poor**. As in the case of the first pathway (Fig. 9), the predictive power using 35 instances is minimal. The low production for this pathway (Fig. 2 in Opgenorth et al.[13]) preempted a second cycle. R^2 and Mean Absolute Error (MAE) values are only for cross-validated mean predictions (black data points). Bars indicate 95% credible interval of the predictive posterior distribution. Source data are provided as a Source Data file.

Supplementary References

(1) Kendall, A.; Gal, Y. What Uncertainties Do We Need in Bayesian Deep Learning for Computer Vision? NIPS'17 Proceedings of the 31st Conference on Neural Information Processing Systems. 2017.

(2) Kiureghian, A. D.; Ditlevsen, O. Aleatory or epistemic? Does it matter? *Structural Safety* **2009**, *31*, 105–112.

(3) Breiman, L. Stacked regressions. *Machine Learning* **1996**, *24*, 49–64.

(4) Breiman, L. Bagging Predictors. *Machine Learning* **1996**, *24*, 123–140.

(5) Freund, Y.; Schapire, R. E. A Decision-Theoretic Generalization of On-Line Learning and an Application to Boosting. *Journal of Computer and System Sciences* **1997**, *55*, 119–139.

(6) Hoeting, J. A.; Madigan, D.; Raftery, A. E.; Volinsky, C. T. Bayesian model averaging: a tutorial. *Statistical Science* **1999**, *14*, 382–417.

(7) Lacoste, A.; Marchand, M.; Laviolette, F.; Larochelle, H. Agnostic Bayesian Learning of Ensembles. Proceedings of the 31st International Conference on Machine Learning. 2014; pp 611–619.

(8) Monteith, K.; Carroll, J. L.; Seppi, K.; Martinez, T. Turning Bayesian model averaging into Bayesian model combination. The 2011 International Joint Conference on Neural Networks. 2011.

(9) Chipman, H. A.; George, E. I.; McCulloch, R. E. Bayesian Ensemble Learning. Proceedings of the 19th International Conference on Neural Information Processing Systems. 2006; pp 265–272.

(10) Yao, Y.; Vehtari, A.; Simpson, D.; Gelman, A. Using Stacking to Average Bayesian Predictive Distributions (with Discussion). *Bayesian Analysis* **2018**, *13*, 917–1003.

(11) van Rossum, G.; Warsaw, B.; Coghlan, N. Style Guide for Python Code. 2001; `www.python.org/dev/peps/pep-0008/`.

(12) Zhang, J.; Petersen, S. D.; Radivojevic, T.; Ramirez, A.; Perez, A.; Abeliuk, E.; Sanchez, B. J.; Costello, Z.; Chen, Y.; Fero, M., et al. Predictive engineering and optimization of tryptophan metabolism in yeast through a combination of mechanistic and machine learning models. *BioRxiv* **2019**, 858464.

(13) Opgenorth, P. et al. Lessons from Two Design–Build–Test–Learn Cycles of Dodecanol Production in Escherichia coli Aided by Machine Learning. *ACS Synth. Biol.* **2019**, *8*, 1337–1351.

(14) McKay, M. D.; Beckman, R. J.; Conover, W. J. A Comparison of Three Methods for Selecting Values of Input Variables in the Analysis of Output from a Computer Code. *Technometrics* **1979**, *21*, 239–245.

(15) Weber, E.; Engler, C.; Gruetzner, R.; Werner, S.; Marillonnet, S. A modular cloning system for standardized assembly of multigene constructs. *PloS one* **2011**, *6*.
